# Oligodendrocytes Are Active Participants in the Pathogenesis of Multiple Sclerosis and Its Animal Models

**DOI:** 10.3390/ijms27041779

**Published:** 2026-02-12

**Authors:** Min Li Lin, Wensheng Lin

**Affiliations:** 1 Department of Neuroscience, University of Minnesota, Minneapolis, MN 55455, USA; minlin8888@hotmail.com; 2 Institute for Translational Neuroscience, University of Minnesota, Minneapolis, MN 55455, USA

**Keywords:** multiple sclerosis, experimental autoimmune encephalomyelitis, oligodendrocyte, myelin, demyelination, intrinsic oligodendrocyte defect

## Abstract

Multiple sclerosis (MS) and its animal model experimental autoimmune encephalomyelitis (EAE) are autoimmune inflammatory demyelinating diseases of the central nervous system (CNS). For decades, oligodendrocytes were regarded as passive targets of autoimmune inflammation in these conditions. However, recent studies challenge this view, revealing that oligodendrocytes are active participants—not just passive targets—in the pathogenesis of MS and EAE. In this review, we summarize recent research that highlights the active and dynamic roles of oligodendrocytes in these diseases.

## 1. Introduction

Multiple sclerosis (MS) is a chronic inflammatory demyelinating disease of the central nervous system (CNS) that primarily affects young adults, typically between the ages of 20 and 40, and is more prevalent in women [1,2,3]. The clinical presentation and progression of MS are highly variable [1,2,3]. Approximately 85% of patients are initially diagnosed with the relapsing–remitting form (RRMS), characterized by episodes of neurological dysfunction followed by periods of recovery. Over time, RRMS often transitions into secondary progressive MS (SPMS), which is marked by steadily worsening and irreversible neurological deficits. A smaller subset of patients experience a progressive decline from disease onset, known as primary progressive MS (PPMS).

MS is believed to result from a complex interplay between genetic susceptibility and environmental risk factors [2,3,4,5,6]. A variety of environmental factors may contribute to the development of MS, particularly during critical time periods. These include limited sun exposure, low vitamin D levels, and certain viral infections, among others [2,3,4,5,6]. Importantly, recent studies have suggested that Epstein–Barr virus is a major causal factor in MS, particularly in young patients, by inducing autoimmunity against myelin [7,8]. Genetic predisposition is strongly supported by familial aggregation data [9,10,11,12]. For instance, the age-adjusted risk of MS is approximately 35% in monozygotic twins, compared to 6% in dizygotic twins and 3% in non-twin siblings—highlighting the role of shared genetic factors. MS heritability is polygenic, involving numerous genetic polymorphisms, each conferring a modest increase in disease risk [9,10,11,12]. Among these, variations in the human leukocyte antigens (HLA) class I and class II genes have the strongest association with MS susceptibility. Genome-wide association studies (GWASs) have identified over 233 genetic variants linked to MS risk. While each variant individually has a small effect, their cumulative and combinatorial impact likely contributes to the overall genetic susceptibility in individual patients [9,10,11,12].

The hallmark of MS pathology is the presence of demyelinating plaques in the white and gray matter of the brain and spinal cord. These plaques are characterized by inflammation, oligodendrocyte loss, demyelination, and axon degeneration [1,2,3]. Oligodendrocytes are primarily responsible for producing myelin, a multilayered membrane that wraps concentrically around axons and enables saltatory conduction of action potentials [13,14,15]. Myelin is essential for the precise synchronization of action potentials, which is critical for integrating excitatory and inhibitory inputs and ensuring accurate timing of neuronal communication [16,17,18]. Damage to myelin disrupts this synchronization, impairing neural circuit function and contributing to the wide range of neurological symptoms observed in MS. In addition to facilitating signal transmission, oligodendrocytes and myelin provide metabolic support for axons and help protect them from various insults [19,20,21]. Demyelination is widely regarded as a major contributor to axonal degeneration in MS, although the underlying mechanisms remain incompletely understood [20,22].

**Figure 1:**
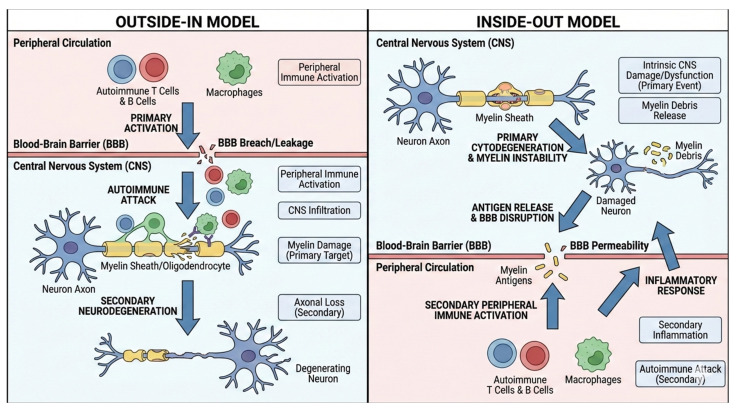
The etiopathogenesis of MS: the “outside–in” model vs. the “inside–out” model. In the ‘outside–in’ model, autoimmunity against myelin is primed in the peripheral immune system; autoreactive lymphocytes then migrate into the CNS, where they initiate autoimmune inflammation targeting myelin and oligodendrocytes. In the ‘inside–out’ model, primary damage to oligodendrocytes and myelin leads to the release of myelin antigens, which activate autoreactive lymphocytes. These lymphocytes subsequently infiltrate the CNS, driving secondary autoimmune inflammation and exacerbating demyelination.

The etiopathogenesis of MS remains a subject of debate, with two primary hypotheses proposed: the “outside–in” and “inside–out” models (Figure 1) [23,24,25,26]. The “outside–in” hypothesis posits that MS begins with the generation of myelin-reactive T cells in the peripheral immune system through unknown mechanisms. These myelin-reactive T cells migrate into the CNS, where they initiate autoimmune inflammation targeting myelin and oligodendrocytes. The resulting damage further amplifies autoimmune inflammation, creating a self-sustaining cycle of inflammation and tissue injury. In contrast, the “inside–out” hypothesis suggests that MS originates within the CNS, beginning with primary damage to oligodendrocytes and myelin caused by unidentified triggers. This initial injury leads to the release of myelin antigens, which activate autoreactive T cells. These myelin-reactive T cells subsequently infiltrate the CNS, driving autoimmune inflammation and causing additional demyelination and oligodendrocyte injury. Although these models differ in the proposed initiating event, both converge on a common pathway characterized by immune-mediated demyelination [23,24,25,26].

Traditionally, under the “outside–in” hypothesis, oligodendrocytes and myelin have been viewed as passive targets of autoimmune inflammation in MS [1,2,3]. However, recent studies challenge this notion, suggesting that oligodendrocytes actively participate in the pathogenesis of MS and experimental autoimmune encephalomyelitis (EAE), a widely used animal model that supports the “outside–in” framework of MS [27,28,29,30,31,32,33,34,35]. It has been shown that oligodendrocytes can function as active immunomodulators, influencing the development of MS and EAE [29,30,31,35]. Gain- and loss-of-function genetic studies have shown that intrinsic oligodendrocyte defects caused by genetic manipulations increase susceptibility to EAE in mice by promoting oligodendrocyte death and/or inflammation [32]. More strikingly, the “inside–out” hypothesis posits that oligodendrocyte death and myelin damage are not merely consequences but essential initiators of autoimmune inflammation in MS [23,24,25,26]. In this review, we summarize recent research highlighting the active and dynamic roles of oligodendrocytes in the pathogenesis of MS and EAE.

## 2. The “Inside-Out” Hypothesis Proposes That Oligodendrocyte Death and Myelin Damage Are Essential Initiators of Autoimmune Inflammation in MS

The success of the EAE model—which supports the “outside–in” hypothesis—has led to the development of various immunosuppressive and immunomodulatory therapies for MS [36,37,38,39,40,41,42]. These anti-inflammatory treatments have proven highly effective in suppressing peripherally driven inflammation and reducing relapses in RRMS. However, their impact on the accumulation of irreversible tissue damage, which is the main cause of progressive neurological decline, remains limited. Furthermore, these treatments offer little to no benefit for patients with progressive MS [39,40,41,42]. Additionally, it has been shown that many RRMS patients display “silent progression”, characterized by a gradual decline in neurological function without clinical relapses or detectable inflammatory activity on MRI [43,44]. These findings raise the possibility that CNS tissue damage—specifically oligodendrocyte death and myelin degeneration—may be driven by non-inflammatory mechanisms. More strikingly, pathological studies reveal that oligodendrocyte death is the earliest structural change in newly formed MS lesions, occurring prior to the onset of inflammation [23,27,28]. Using CNS tissue from a young RRMS patient who died within 24 h of symptom onset, a study reports that the earliest structural change in newly formed brainstem lesions is extensive oligodendrocyte apoptosis [27]. The lesions also display early microglial activation but minimal lymphocyte infiltration or myelin phagocytes. Similar pathological features were identified in nine additional lesions from 6 other patients within the 11-patient cohort of rapidly progressive MS [27]. In another study analyzing 26 active lesions from 11 patients (within the 11-patient cohort) with early-stage MS, oligodendrocyte apoptosis is consistently observed at the borders of rapidly expanding lesions [28]. These regions display largely intact myelin, activated microglia, and limited lymphocyte presence. In contrast, recently demyelinated tissue contains numerous foamy macrophages and a large infiltration of lymphocytes [28]. These pathological findings challenge the ‘outside–in’ hypothesis and highlight an alternative perspective on MS pathogenesis—the ‘inside–out’ hypothesis [23,24]. This model proposes that oligodendrocyte death and myelin damage, driven by non-inflammatory mechanisms, are the primary initiating events that trigger autoimmune inflammation in MS [23,24,25,26].

The first animal model supporting the “inside–out” hypothesis involves diphtheria toxin (DTA)-mediated ablation of oligodendrocytes. In this model, Traka et al. employ *Plp1-CreER^T^;ROSA26-eGFP-DTA* mice, where tamoxifen administration triggers DTA expression specifically in oligodendrocytes [45]. This results in widespread oligodendrocyte death, severe CNS demyelination, and severe neurological impairment. Interestingly, by 10 weeks post injection, these mice show marked clinical recovery, associated with oligodendrocyte regeneration and remyelination. However, around 40 weeks after the initial insult, these mice develop a fatal secondary disease marked by extensive loss of myelin and axons and infiltration of T lymphocytes in the CNS as well as the presence of myelin oligodendrocyte glycoprotein (MOG)35-55-autoreactive T lymphocytes in lymphoid organs. Importantly, inducing MOG35–55-specific tolerance prevents the development of the fatal secondary disease in these mice. Moreover, adoptive transfer of these MOG35–55-autoreactive T cells into naïve mice induces neurological symptoms resembling EAE, although milder, along with demyelination and T lymphocyte-mediated inflammation in the CNS white matter [45]. This study provides strong evidence that oligodendrocyte death in the CNS can trigger adaptive autoimmunity against myelin in the peripheral and results in immune-mediated demyelinating disease in the CNS, thus lending robust support to the “inside–out” hypothesis.

The cuprizone autoimmune encephalitis (CAE) model is another experimental system that supports the “inside–out” hypothesis [46]. The cuprizone model has been widely used to investigate the mechanisms of demyelination and remyelination in the CNS [47,48,49]. Cuprizone is believed to directly target oligodendrocytes, inducing their apoptosis and leading to subsequent demyelination. While innate immunity is involved in demyelination and remyelination in this model, the involvement of adaptive immunity has been ruled out [47,48,49]. In the CAE model developed by Caprariello et al., adult mice are treated with cuprizone for two weeks, followed by an artificial immune boost using complete Freund’s adjuvant and pertussis toxin [46]. Notably, two weeks after the immune boost, these mice develop inflammatory demyelinating lesions in the CNS, accompanied by the presence of myelin-autoreactive T lymphocytes in the spleen [46]. On the other hand, oligodendrocyte death and myelin damage occur in various neurological diseases, including traumatic brain injury, stroke, and hereditary CNS demyelinating diseases, among others [50,51,52]. In most cases, however, secondary anti-myelin autoimmunity does not develop. Similarly, in various demyelinating animal models, primary oligodendrocyte death induced by genetic mutations or neurotoxins does not lead to secondary anti-myelin autoimmunity [52,53,54,55]. Collectively, these findings suggest that oligodendrocyte death and myelin damage can trigger adaptive autoimmunity against myelin only when accompanied by a permissive immune environment.

## 3. The “Outside-In” Hypothesis: The Intrinsic Vulnerability of Oligodendrocytes Determines Susceptibility to MS and EAE

MS demyelinating lesions are marked by inflammation, oligodendrocyte loss, demyelination, and axon degeneration. The EAE model mirrors many key features of MS, including clinical manifestations, pathological changes, and autoimmune responses [36,37,38]. The “outside-in” hypothesis proposes that MS and EAE are initiated by oligodendrocytes and myelin attacked by autoimmune inflammation. The resulting damage further amplifies autoimmune inflammation, creating a self-sustaining cycle of inflammation and tissue injury [24,25,26,32]. Numerous studies have shown that the intrinsic vulnerability of oligodendrocytes to autoimmune attack determines susceptibility to MS and EAE.

### 3.1. Intrinsic Apoptotic Signaling in Oligodendrocytes Influences Susceptibility to MS and EAE

Blocking apoptotic signaling in oligodendrocytes has been shown to reduce susceptibility to EAE in mice [56,57,58,59,60]. Hisahara et al. demonstrate the presence of caspase-11 and activated caspase-3 in oligodendrocytes within demyelinated regions of EAE mice [57]. Notably, caspase-11-deficient oligodendrocytes are resistant to immune cytokine-induced apoptosis. Caspase-11-deficient mice also exhibit resistance to EAE, with reduced oligodendrocyte apoptosis and CNS inflammation [57]. Furthermore, Hisahara et al. show that overexpression of the anti-apoptotic protein p35 in oligodendrocytes protects them from immune cytokine-induced cytotoxicity in vitro. In vivo, oligodendrocyte-specific expression of p35 similarly confers protection against EAE, with decreased oligodendrocyte apoptosis and inflammation [58]. Hovelmeyer et al. generate mice with oligodendrocyte-specific deletion of death receptor Fas, TNF-R1 (tumor necrosis factor receptor 1), or both receptors. Deletion of either receptor individually leads to milder EAE symptoms, while double deletion results in minimal clinical signs after EAE induction. This is accompanied by reduced oligodendrocyte apoptosis, demyelination, and inflammation in the CNS of EAE mice [59]. Similarly, McGuire et al. develop mice with oligodendrocyte-specific deletion of FADD (Fas-associated death domain protein), a key mediator linking death receptors to caspase activation. In vitro, FADD-deficient oligodendrocytes are resistant to death receptor-mediated apoptosis. In the EAE model, FADD deletion in oligodendrocytes significantly ameliorates EAE disease severity and reduces demyelination and inflammation in the CNS [60]. Collectively, these findings suggest that modulating intrinsic apoptotic signaling in oligodendrocytes can significantly influence susceptibility to MS and EAE.

### 3.2. The Unfolded Protein Response (UPR) in Oligodendrocytes Influences Susceptibility to MS and EAE

**Figure 2:**
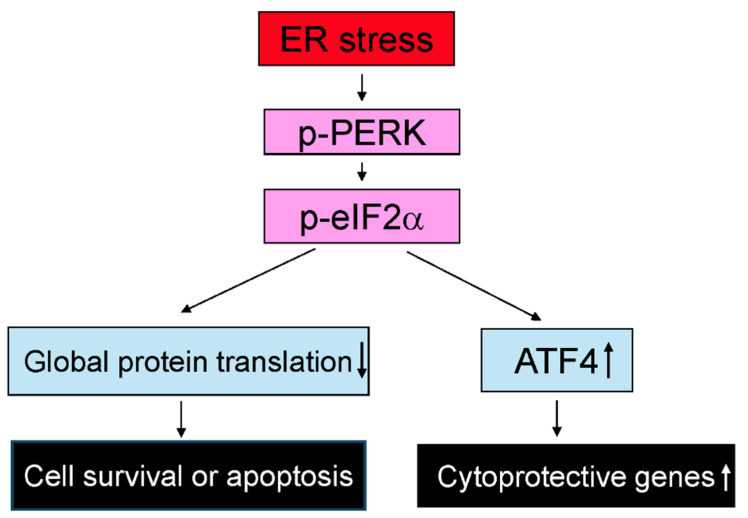
The PERK–eIF2α pathway. ER stress induces PERK autophosphorylation, leading to phosphorylation of eIF2α. Phosphorylated eIF2α suppresses global protein translation while selectively promoting the expression of cytoprotective genes through induction of ATF4.

The UPR, triggered by endoplasmic reticulum (ER) stress, plays a critical role in regulating cell viability and function under both physiological and pathological conditions [61,62]. Oligodendrocytes are particularly sensitive to disruptions in ER homeostasis, and the UPR is a key regulator of their survival and function in both health and disease [63,64,65]. In MS and EAE, activation of the UPR has been observed in oligodendrocytes [63,64]. It has been shown that activation of the UPR, particularly the pancreatic ER kinase (PERK) branch, in oligodendrocytes affects their viability and influences susceptibility to EAE in mice [63,64]. PERK activation promotes the expression of cytoprotective genes while inhibiting global protein translation through phosphorylation of eukaryotic translation initiation factor 2α (eIF2α) (Figure 2) [61,62,63,64,65]. A study shows that CNS expression of interferon-γ (IFN-γ) prior to EAE onset protects mice against the disease, accompanied by reduced oligodendrocyte apoptosis and demyelination, along with activation of the PERK-eIF2α pathway in oligodendrocytes. Notably, global PERK heterozygous knockout diminishes the protective effects of IFN-γ in EAE, suggesting a cytoprotective role for PERK activation in oligodendrocytes [66]. This notion is further supported by oligodendrocyte-specific conditional mouse models. A study shows that deletion of PERK specifically in oligodendrocytes increases susceptibility to EAE by promoting oligodendrocyte apoptosis and demyelination [67]. Accordingly, another study shows that enhancing activation of the PERK-eIF2α pathway specifically in oligodendrocytes reduces susceptibility to EAE by preventing oligodendrocyte apoptosis and demyelination [68]. Moreover, pharmacological agents such as Guanabenz and Sephin1, which inhibit dephosphorylation of phosphorylated eIF2α, enhance the activity of the PERK-eIF2α pathway in oligodendrocytes and ameliorate EAE severity, oligodendrocyte loss and demyelination [69,70]. Interestingly, deletion of activating transcription factor 4 (ATF4), a major transcription factor downstream of phosphorylated eIF2α, in oligodendrocytes does not affect EAE severity, oligodendrocyte apoptosis or demyelination, suggesting that not all components of the PERK pathway contribute equally to its protective effects [71].

On the other hand, recent studies suggest that PERK activation is not exclusively beneficial to oligodendrocytes [16,65,72,73,74]. While it can protect against apoptosis, PERK activation also suppresses the myelinating function of oligodendrocytes by inhibiting the translation of myelin proteins under both normal and disease conditions [16,65,72,73,74]. In EAE demyelinating lesions, PERK activation in oligodendrocytes reduces their apoptosis and demyelination (a destructive form of myelin loss) but induces thinning of originally existing, mature myelin (a nondestructive form of myelin loss) by inhibiting myelin protein translation [16,68]. In addition to the PERK-eIF2α branch, the activating transcription factor 6 (ATF6)-binding immunoglobulin protein (BiP) branch of the UPR has also been implicated in oligodendrocyte protection. Global ATF6 knockout increases EAE severity and worsens oligodendrocyte apoptosis and demyelination [75]. Similarly, oligodendrocyte-specific deletion of BiP leads to more severe EAE and exacerbates oligodendrocyte loss and demyelination [76]. Take together, these studies imply that the UPR in oligodendrocytes influences susceptibility to MS and EAE by altering the vulnerability of oligodendrocytes to autoimmune inflammation.

### 3.3. Nuclear Factor-κB (NF-κB) Signaling in Oligodendrocytes Influences Susceptibility to MS and EAE

The transcription factor NF-κB is a critical player in regulating inflammation and cell viability in various inflammatory diseases, including MS and EAE [77,78,79,80]. NF-κB functions as a homo- or heterodimer composed of members of the Rel family (including p65, c-Rel, RelB, p50, and p52) and is normally held inactive in the cytoplasm through binding to inhibitory IκB (inhibitor of κB) proteins. Upon release of IκBs, NF-κB translocates to the nucleus to activate the transcription of target genes [77,78,79,80].

NF-κB activation in oligodendrocytes has been well documented in both MS and EAE [68,79,80,81,82]. In vitro studies show that NF-κB activation protects oligodendrocytes from the cytotoxic effects of inflammatory mediators [83,84,85,86]. Using a mouse model expressing IκBαΔN (a dominant-negative form of IκBα with an N-terminal deletion that inhibits NF-κB activation) specifically in oligodendrocytes, a study shows that blocking NF-κB activation in oligodendrocytes via overexpression of IκBαΔN increases their sensitivity to IFN-γ in both young, developing mice and in the cuprizone model of demyelination [87]. Notably, oligodendrocyte-specific overexpression of IκBαΔN results in extremely severe EAE, with most mice dying suddenly at disease onset [87]. While this finding suggests the possibility that blocking NF-κB activation in oligodendrocytes via overexpression of IκBαΔN promotes their death and subsequently results in increased EAE disease severity, the sudden mortality limits definitive conclusions. Moreover, using a mouse model that expresses a constitutively active form of inhibitor of NF-κB kinase 2 (IKK2ca, which activates NF-κB through IKK2-dependent canonical pathway) specifically in oligodendrocytes, a study demonstrates that enhanced NF-κB activation in oligodendrocytes via overexpression of IKK2ca does not affect oligodendrocyte viability or functions under normal conditions; however, it protects mice against EAE, accompanied by reduced oligodendrocyte death and demyelination [82]. This study further shows that the cytoprotective effects of NF-κB activation on oligodendrocytes during EAE are associated with upregulation of A20/TNFAIP3 (tumor necrosis factor α-induced protein 3), a NF-κB-responsive anti-apoptotic genes [82].

Interestingly, A20/TNFAIP3 is considered an MS risk gene. Polymorphisms in A20/TNFAIP3, associated with reduced function or expression of the A20/TNFAIP3 protein, increase susceptibility to MS [80,88,89,90]. Using a mouse model with A20/TNFAIP3 specifically deleted in mature oligodendrocytes, our unpublished data show that this deletion does not affect oligodendrocyte viability or function in adult naïve mice, but significantly exacerbates disease severity, oligodendrocyte death, and demyelination in the EAE model (unpublished data). In contrast, a separate study reports that IKK2 deletion in oligodendrocytes has a minimal effect on EAE progression, although it does not provide evidence that IKK2 deletion alone is sufficient to abolish NF-κB activation in oligodendrocytes during EAE [91]. Taken together, these studies suggest that activation of NF-κB signaling in oligodendrocytes reduces susceptibility to MS and EAE by protecting oligodendrocytes from autoimmune inflammation.

### 3.4. IFN-γ Signaling in Oligodendrocytes Influences Susceptibility to MS and EAE

The immune cytokine IFN-γ plays a central role in regulating inflammation and cell viability in various inflammatory diseases, including MS and EAE [92,93,94]. IFN-γ exerts its effects through its receptors, IFN-γR1 and IFN-γR2, which activate Janus kinases (JAKs) and signal transducer and activator of transcription 1 (STAT1), leading to the upregulation of IFN-γ-responsive genes, such as interferon regulatory factor 1 (IRF-1). Conversely, suppressor of cytokine signaling 1 (SOCS1) inhibits IFN-γR–JAK/STAT1 signaling and modulates the cellular effects of IFN-γ [92,93,94].

In vitro and in vivo studies have demonstrated that IFN-γ regulates oligodendrocyte viability through IFN-γR–JAK/STAT1 signaling [95,96]. Moreover, it has been shown that IFN-γ influences susceptibility to MS and EAE by regulating oligodendrocyte viability through IFN-γR–JAK/STAT1 signaling. Mice lacking IFN-γ or its receptors exhibit increased susceptibility to EAE compared to wild-type controls [97,98,99]. CNS-specific overexpression of IFN-γ prior to cuprizone treatment protects mature oligodendrocytes and myelin from cuprizone-induced neurotoxicity [100]. Similarly, CNS-specific overexpression of IFN-γ prior to EAE onset protects mature oligodendrocytes and myelin against inflammation by activating PERK signaling in oligodendrocytes, thereby reducing EAE disease severity [66]. Moreover, oligodendrocyte-specific overexpression of SOCS1 inhibits their response to IFN-γ and leads to exacerbation of oligodendrocyte apoptosis, demyelination, and disease severity in the EAE model [101]. Interestingly, IRF-1 has been identified as an MS risk gene [102]. IRF-1 knockout mice appear normal but are resistant to EAE [103,104]. oligodendrocyte-specific expression of dominant-negative IRF-1 (dnIRF-1) attenuates oligodendrocyte apoptosis and demyelination and results in decreased disease severity in EAE model [105]. Collectively, these findings suggest that IFN-γR–JAK/STAT–IRF-1 signaling in oligodendrocytes influences susceptibility to MS and EAE by modulating oligodendrocyte vulnerability to autoimmune inflammation.

### 3.5. Additional Studies Show the Critical Role of Oligodendrocytes in Determining Susceptibility to EAE

Additional studies using oligodendrocyte-specific conditional knockout mouse models further highlight the critical role of oligodendrocytes in determining susceptibility to EAE. Saldivia et al. report that deletion of galactosylceramidase (GALC) specifically in mature oligodendrocytes exacerbates demyelination and increases disease severity in the EAE model [106]. Madsen et al. demonstrate that deletion of tumor necrosis factor receptor 2 (TNFR2) specifically in oligodendrocytes worsens demyelination and disease severity in the EAE model [107]. In contrast, Locatelli et al. find that deletion of insulin-like growth factor 1 receptor (IGF1R) in mature oligodendrocytes attenuates EAE severity [108]. Rajendran et al. show that deletion of fibroblast growth factor receptor 1 (FGFR1) specifically in mature oligodendrocytes results in reduced demyelination and disease severity in the EAE model [109]. Kamali et al. report that deletion of fibroblast growth factor receptor 2 (FGFR2) specifically in mature oligodendrocytes leads to the attenuation of demyelination and disease severity in the EAE model [110].

## 4. Oligodendrocytes Function as Active Immunomodulators, Influencing the Development of MS and EAE

In addition to their primary role in producing myelin, oligodendrocytes also express molecules involved in antigen presentation, such as major histocompatibility complex (MHC) class I and II, co-stimulatory molecules, and a range of inflammatory mediators, including cytokines, chemokines, and components of the complement system and its receptors [29,30,31,35]. Growing evidence suggests that oligodendrocytes actively participate in regulating autoimmune inflammation in MS and EAE.

Recent studies suggest that oligodendrocyte lineage cells regulate autoimmune inflammation in MS and EAE by functioning as antigen-presenting cells. In vitro studies have shown that IFN-γ stimulates the expression of MHC molecules in oligodendrocyte lineage cells [111,112,113,114]. In vivo, transgenic mouse models demonstrate that IFN-γ overexpression in the CNS induces MHC expression in oligodendrocytes via the JAK/STAT signaling pathway [95,96,115]. Single-cell and single-nucleus RNA sequencing data further reveal that oligodendrocyte lineage cells express antigen presentation molecules in MS and EAE [114,116,117]. The use of MHC class I and II reporter mice confirms MHC expression in oligodendrocytes during EAE [118]. Evidence suggests that oligodendrocytes can function as antigen-presenting cells in the context of MS and EAE. One study demonstrates that oligodendrocytes can express myelin basic protein (MBP)-MHC complexes and present MBP peptides to MBP-specific CD8^+^ T cells during EAE [119]. Moreover, immunization with ovalbumin (OVA) induces EAE in MBP-OVA transgenic mice that express OVA in oligodendrocytes, indicating their ability to present antigens and activate T cells [120]. MBP-OVA/OT-I double-transgenic mice, which express OVA in oligodendrocytes and harbor OVA-specific CD8^+^ T cells, develop spontaneous EAE [121]. In contrast, MBP-OVA/OT-II mice, which express OVA in oligodendrocytes and harbor OVA-specific CD4^+^ T cells, do not develop spontaneous disease. These results suggest that oligodendrocytes primarily present antigens to CD8^+^ rather than CD4^+^ T cells [121]. Additionally, adoptive transfer of hemagglutinin (HA)-specific CD8^+^ effector T cells into MOG-HA mice, which express HA in oligodendrocytes, also induces EAE [122]. On the other hand, evidence also suggests that oligodendrocyte precursor cells (OPCs) can function as antigen-presenting cells in MS and EAE [113]. IFN-γ has been shown to induce antigen-presenting capacity in OPCs by promoting immunoproteasome expression. IFN-γ-conditioned OPCs upregulate MHC class I expression and are capable of presenting antigen to CD8^+^ T cells both in vitro and in vivo [113]. Consistently, analysis of postmortem MS tissue shows immunoproteasome-expressing OPCs within white matter lesions, suggesting that these phenotypic changes also occur in human disease [113].

Several lines of evidence suggest that oligodendrocytes contribute to the regulation of inflammation in MS and EAE through the production of cytokines. Tzartos et al. report that oligodendrocytes produce IL-17A (interleukin-17A), a pro-inflammatory cytokine that enhances immune responses by acting on both immune and non-immune cells [123]. Cannella and Raine find that oligodendrocytes express IL-18 (interleukin-18), which promotes inflammation by stimulating IFN-γ production [124]. Ma et al. show that oligodendrocytes produce IL-6 (interleukin-6), which can recruit immune cells to sites of inflammation and influences the development and function of T and B cells [125]. Moreover, several studies have demonstrated that oligodendrocytes produce brain-derived neurotrophic factor (BDNF), which exerts anti-inflammatory effects by directly modulating microglial activity [126,127]. Notably, a recent study highlights the crucial role of oligodendrocyte-derived interleukin-33 (IL-33) in chronic CNS autoimmunity [128]. The researchers show that oligodendrocytes produce IL-33 and use a genetic approach to explore its function. By crossing oligodendrocyte-specific IL-33 knockout mice with MOG-GP mice—which express the lymphocytic choriomeningitis virus glycoprotein (LCMV-GP) as a neo–self-antigen in oligodendrocytes—they demonstrate that IL-33 from oligodendrocytes is essential for modulating the pathogenicity of self-reactive CD8^+^ T cells. Deletion of IL-33 specifically from neo-self-antigen-expressing oligodendrocytes alleviates CNS disease, resulting in reduced persistence of self-reactive CD8^+^ T cells in the inflamed CNS and impaired formation of TCF-1^low^ effector cells. Similarly, therapeutic IL-33 blockade via locally delivered somatic gene therapy reduces T cell infiltration and improves disease outcomes [128].

Additionally, data indicate that oligodendrocytes can modulate inflammation in MS and EAE by producing chemokines (such as CCL2, CCL5, and CXCL10), complement components and receptors (including C1s, C2, C3, and C1R), and complement regulatory molecules (such as CD46, CD55, and CD59), among others [29,30,31,35].

## 5. Concluding Remarks and Future Perspectives

Oligodendrocytes are responsible for producing vast amounts of myelin proteins and lipids necessary to assemble and sustain myelin in the CNS. Their high metabolic demands make them particularly susceptible to inflammatory damage [32,64,129]. As a result, a long-standing but debated view has been that oligodendrocytes are merely passive victims in autoimmune demyelinating diseases such as MS and EAE [1,2,3,32,64,129]. However, recent studies challenge this notion, revealing that oligodendrocytes are an active player—not just passive targets—in the pathogenesis of MS and EAE (Table 1). Two primary hypotheses have been proposed to explain the etiopathogenesis of MS: the “outside–in” and “inside–out” models. The “inside–out” model posits that oligodendrocyte death and myelin damage—initiated by non-inflammatory, intrinsic mechanisms—trigger autoimmune inflammation. This model is supported by animal studies demonstrating that intrinsic oligodendrocyte death, in the context of a permissive immune environment, can initiate autoimmunity against myelin. Meanwhile, the widely used EAE model aligns with the “outside–in” framework, where peripheral immune activation precedes CNS damage. Notably, studies using EAE have shown that genetic manipulations causing intrinsic oligodendrocyte defects increase disease susceptibility by promoting oligodendrocyte death. These findings suggest that the inherent vulnerability of oligodendrocytes significantly influences susceptibility to MS and EAE. Additionally, emerging evidence indicates that oligodendrocytes can actively modulate immune responses, further implicating them as an active player in MS and EAE pathogenesis.

It has been shown that the intrinsic vulnerability of oligodendrocytes to autoimmune attack determines susceptibility to MS and EAE. GWASs have identified over 233 genes linked to an increased risk of MS. While most current research focuses on the autoimmune and inflammatory mechanisms mediated by these risk genes, emerging evidence suggests that some also contribute to MS pathogenesis by directly affecting oligodendrocytes and other CNS cell types [11,12,130,131,132]. There is a critical need to investigate how these risk genes impact oligodendrocyte function, as intrinsic defects in oligodendrocytes driven by genetic polymorphisms can play a direct role in increasing MS susceptibility. Understanding these mechanisms could provide novel insights into the etiology of MS and pave the way for developing therapies that protect oligodendrocytes in MS patients.

While recent studies suggest that oligodendrocyte lineage cells may function as antigen-presenting cells, the mechanisms underlying antigen presentation by these cells remain poorly understood. The functional consequences of antigen presentation by oligodendrocyte lineage cells are also unclear. Furthermore, it is not yet known whether their antigen-presenting capabilities differ functionally from those of classical antigen-presenting cells. Addressing these open questions is crucial for advancing our understanding of their role in MS.

Although there is evidence that oligodendrocytes can produce a variety of inflammatory mediators, including cytokines and chemokines, among others, the functional significance of these oligodendrocyte-derived inflammatory mediators in the context of MS remains largely unknown. Investigating their roles using oligodendrocyte-specific conditional knockout or transgenic mouse models is essential for understanding how these oligodendrocyte-derived mediators contribute to MS pathogenesis.

## Figures and Tables

**Table 1:** The Paradigm Shift: active and dynamic roles of oligodendrocytes in the pathogenesis of MS and EAE.

	The Traditional View	The Current View
**Oligodendrocytes in MS and EAE**	Merely passive victims of autoimmune inflammation	Active participants in disease pathogenesis
**Active function of oligodendrocytes in MS and EAE**	n/a	Determinants of disease susceptibility
	n/a	Active modulators of autoimmune inflammation

## Data Availability

All data generated or analyzed in this study are included in this paper and can be obtained from the authors upon reasonable request.
